# Towards accurate partial volume correction in ^99m^Tc oncology SPECT: perturbation for case-specific resolution estimation

**DOI:** 10.1186/s40658-022-00489-5

**Published:** 2022-09-05

**Authors:** Rebecca Gillen, Kjell Erlandsson, Ana M. Denis-Bacelar, Kris Thielemans, Brian F. Hutton, Sarah J. McQuaid

**Affiliations:** 1 Institute of Nuclear Medicine, University College London, London, UK; 2 National Physical Laboratory, Teddington, UK; 3 Department of Clinical Physics and Bioengineering, Nuclear Medicine, North East Sector, NHS Greater Glasgow and Clyde, Glasgow, UK; 4 Centre for Medical Image Computing, University College London, London, UK

**Keywords:** Perturbation, SPECT, Quantification, Partial volume correction

## Abstract

**Background:**

Currently, there is no consensus on the optimal partial volume correction (PVC) algorithm for oncology imaging. Several existing PVC methods require knowledge of the reconstructed resolution, usually as the point spread function (PSF)—often assumed to be spatially invariant. However, this is not the case for SPECT imaging. This work aimed to assess the accuracy of SPECT quantification when PVC is applied using a case-specific PSF.

**Methods:**

Simulations of SPECT $^{99{\mathrm{m}}}$Tc imaging were performed for a range of activity distributions, including those replicating typical clinical oncology studies. Gaussian PSFs in reconstructed images were estimated using perturbation with a small point source. Estimates of the PSF were made in situations which could be encountered in a patient study, including; different positions in the field of view, different lesion shapes, sizes and contrasts, noise-free and noisy data. Ground truth images were convolved with the perturbation-estimated PSF, and with a PSF reflecting the resolution at the centre of the field of view. Both were compared with reconstructed images and the root-mean-square error calculated to assess the accuracy of the estimated PSF. PVC was applied using Single Target Correction, incorporating the perturbation-estimated PSF. Corrected regional mean values were assessed for quantitative accuracy.

**Results:**

Perturbation-estimated PSF values demonstrated dependence on the position in the Field of View and the number of OSEM iterations. A lower root mean squared error was observed when convolution of the ground truth image was performed with the perturbation-estimated PSF, compared with convolution using a different PSF. Regional mean values following PVC using the perturbation-estimated PSF were more accurate than uncorrected data, or data corrected with PVC using an unsuitable PSF. This was the case for both simple and anthropomorphic phantoms. For the simple phantom, regional mean values were within 0.7% of the ground truth values. Accuracy improved after 5 or more OSEM iterations (10 subsets). For the anthropomorphic phantoms, post-correction regional mean values were within 1.6% of the ground truth values for noise-free uniform lesions.

**Conclusion:**

Perturbation using a simulated point source could potentially improve quantitative SPECT accuracy via the application of PVC, provided that sufficient reconstruction iterations are used.

## Background

Limited spatial resolution in PET and SPECT imaging leads to quantitative inaccuracies when measuring activity concentration of small objects. This is known as the partial volume effect (PVE). A range of partial volume correction (PVC) methods have been proposed in the literature [1], several of which depend on knowledge of the reconstructed resolution, often expressed as the Point Spread Function (PSF). The PSF is usually assumed to be position invariant, but this is not always the case, especially in SPECT. Therefore, measuring the case-specific PSF may improve the accuracy of PVC.

### Quantitative SPECT in oncology

Most work on PVC to date has been focused on PET imaging, often for cardiac or brain studies. There is currently no consensus on the most suitable PVC algorithm for SPECT oncology imaging. This application has specific issues to consider.

#### SPECT-specific PVC considerations

While PET has long been considered a quantitative modality, SPECT has lagged behind. Quantitative SPECT is now increasingly in demand with promising potential applications in disease monitoring, imaging for radionuclide therapy, and measuring response to treatment [2, 3]. However, a suitable PVC algorithm is still required [4].

The resolution of SPECT imaging is inferior to PET, therefore inaccuracies due to the PVE are greater. Inaccuracies should also be considered significant for larger objects than in PET (up to 4–5 cm in size for $^{99{\mathrm{m}}}$Tc imaging [3, 5], and approximately 6 cm for some therapy isotopes [6]).

The assumption of invariant PSF may be acceptable in limited situations. However, in SPECT imaging, the distance-dependent detector resolution response means that the PSF depends on the position in the Field of View (FOV) and must be accounted for when applying PVC.

#### Oncology-specific PVC considerations

In oncology imaging, lesions or organs of interest could be in any possible position within the body and across the FOV. This, combined with the distance-dependent detector response, means that the PSF required for accurate PVC will vary on a patient-by-patient, or lesion-by-lesion, basis. In addition, tumours can exist in a wide range of sizes, shapes, and intensities. Tumours may also change in geometry and tracer uptake with disease progression or following treatment.

Therefore, for accurate application of PVC, a suitable method for measuring the PSF must not be limited to specific positions or lesion geometries and should not depend on the target to background ratio (TBR) or lesion size. Other factors relevant to selecting a PVC method suitable for oncology include that the method should not assume a spherical lesion and should not require segmentation of the entire image.

#### Nonlinear reconstruction algorithms and resolution modelling

Iterative reconstruction algorithms, including ordered subset expectation maximisation (OSEM), are the clinical standard for SPECT imaging since they allow attenuation and scatter corrections to be applied. OSEM is a nonlinear reconstruction algorithm, meaning that the reconstructed resolution depends on the activity distribution as well as the number of iterations used. This is another reason that assuming an invariant reconstructed PSF is inappropriate.

Resolution Modelling (RM) algorithms are commonly used in iterative reconstruction in an attempt to compensate for limited resolution, with the aim of reducing quantitative bias due to the PVE. However, it has been shown that the application of resolution modelling does not provide a complete correction for the effect of limited resolution, therefore some PVC is still required [1, 7]. Even if some reduction in bias is seen by applying RM, the variance has been found to increase which limits the precision of quantification [8].

Previous work found that more reconstruction iterations were required than are typically used in clinical practice in order to get a benefit from applying resolution modelling [9]. This work also noted that ringing artefacts can add difficulty in accurately characterising the PSF. These occur when RM is applied, and are more severe at higher reconstruction iterations. Due to these limitations of reconstructing using RM, the data presented in this work has been reconstructed without resolution modelling.

### Perturbation for PSF measurement

A technique which could be used to measure the PSF, for application in PVC, is perturbation. The perturbation method, first demonstrated in 1988 [10], involves adding projections of a small noise-free source to sinogram data, creating a “perturbed” dataset. Following reconstruction, in image space, the original dataset is subtracted from the perturbed dataset. If a point source is used as the perturbation, the resulting difference image represents the PSF for that specific imaging case (specific position in FOV, reconstruction settings, and activity distribution).

Perturbation has been used to demonstrate, through simulations, that the PSF is object dependent for maximum likelihood reconstruction algorithms [10]. Further simulation work on maximum likelihood reconstruction, using a small Gaussian perturbation, concluded that the measured PSF was dependent on the object size and the number of iterations used in the reconstruction [11]. Perturbation has also been used in physical data to evaluate the resolution of a novel SPECT system [12], and to investigate the effect of using resolution modelling in the reconstruction of a SPECT brain phantom, compared with a simple uniform cylinder phantom [13].

For reliable PSF measurement, point source contrast (intensity compared with background) should be small enough to avoid artificial enhancement due to the non-negativity constraint in the reconstruction algorithm. A study on the acceptable point source contrast required for stable full width at half maximum (FWHM) values found a threshold of 0.1 (i.e. the reconstructed peak intensity of the point source with background should be less than 110% of the background activity) [14]. This threshold was adhered to in the present study.

#### Perturbation for oncology SPECT PVC

Previous work on the use of perturbation for resolution measurement has covered a range of equipment, simulation and experimental work, on simple and complex datasets including geometric phantoms and anthropomorphic cardiac and brain phantoms [10–15]. This includes work which uses perturbation in combination with the Geometric Transfer Matrix PVC method in brain SPECT imaging [16]. However, to the best of the authors’ knowledge, none of the previous publications have systematically challenged the perturbation method in scenarios commonly encountered in clinical SPECT oncology imaging. The present study examines the use of perturbation for PSF measurement in phantoms with different lesion sizes, contrasts, and positions, and includes both simple phantoms and anthropomorphic phantoms.

The possibility of improved quantitative accuracy by applying PVC with a perturbation-estimated PSF has not yet been assessed in the literature. The present study aims to evaluate this potential improvement.

## Methods

### Phantom generation

Simple geometric phantoms and anthropomorphic datasets were generated. These generated data in image space are referred to as the Ground Truth (GT).

#### Simple geometric phantoms

**Fig. 1:**
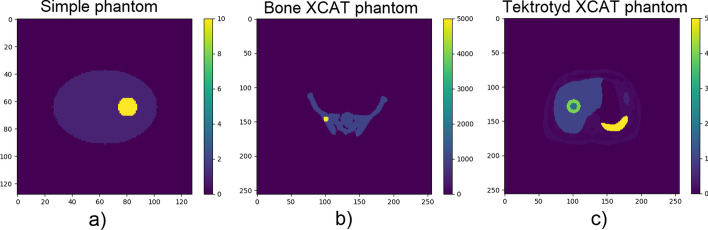
Images showing representative slices of phantoms used in this investigation. **a** The simple geometric phantom with a uniform spherical lesion. **b** Example of an anthropomorphic phantom based on a bone scan, including uniform pelvic lesion, created using XCAT data. **c** Example of an anthropomorphic phantom based on a Tektrotyd scan, including heterogenous liver lesion, created using XCAT data.

A background of a uniform elliptical cylinder (major radius = 152 mm, minor radius = 108 mm, length = 216 mm) was generated on a 128 $\times$ 128 matrix with 4 mm$^3$ voxels. Uniform spheres of higher intensity were added to this uniform background to represent lesions. The different cases examined are summarised in Table 1. An example representative slice of a simple geometric phantom, with a lesion positioned 8 cm from the centre of the FOV (referred to throughout as the isocentre) is shown in Fig. 1a.

For all lesions specified in Table 1, the PSF measurement point was positioned centrally within the lesion. In addition to this, the effect of point position within a lesion was studied. The motivation for this is that, in practice, the exact centre of a lesion may not be known. To perform these measurements, the point position remained at the centre of the FOV. The centre of a 36 mm radius lesion was moved relative to the point along the horizontal axis. This strategy decoupled the effect of the measurement position in the FOV from the effect of the measurement position within a lesion. Measurements at the lesion centre were compared with a point positioned at the edge voxel of the lesion, and a point positioned just outside of the lesion edge (2 voxels away).

**Table 1:** A summary of different lesions examined using the uniform elliptical phantom

Lesion positions	Isocentre, 8 cm from isocentre
Lesion radii	8 mm, 12 mm, 20 mm, 28 mm, 36 mm
Lesion TBRs	5, 10, 25

#### Anthropomorphic phantoms

Anthropomorphic datasets were generated using XCAT software [17] (extended cardiac-torso phantom). Parameters of the XCAT phantom were adjusted to generate a digital phantom which approximately replicated a typical $^{99{\mathrm{m}}}$Tc phosphate scan for bone imaging and a $^{99{\mathrm{m}}}$Tc EDDA/HYNIC-TOC (Tektrotyd) scan used to assess neuroendocrine tumours. The activity distribution for the bone imaging scenario was generated with a small non-zero non-bone-specific background, and the Tektrotyd case was generated with physiological uptake in the liver, spleen, bowel and urinary tract, including kidneys and bladder (relative organ intensities were within ranges observed clinically [18]). Data were generated on $256 \times 256$ matrices with 2.2 mm$^{3}$ voxels. The rationale for using a finer matrix for phantom generation was to attempt to reduce digitisation effects which would not occur in a real clinical situation where the emission source is not voxelised.

The bone scan phantom extent ranged from lumbar spine to the proximal end of the femoral shaft—including the pelvis. Datasets with and without activity in the bladder were generated. Spherical lesions with diameter of 15 mm were added in the right iliac fossa and the right femoral head. An example slice through the pelvis is shown in Fig. 1b. Lesion intensity was set to be five times as intense as normal bone.

The Tektrotyd scan range was mid thorax to bladder. Lesions with different characteristics were added within the liver. Lesions of uniform uptake, diameter 30 mm, with two different intensities were investigated - firstly with intensity 4 times that of normal liver, and also with intensity 6.25 times normal liver (the latter case being 20% more intense than spleen). In addition, a lesion a with non-uniform uptake pattern was generated to simulate a lesion with a necrotic core. The non-uniform lesion had an outer diameter of 44.4 mm, and an inner volume of lower intensity with diameter 22 mm. An example slice through the liver showing the non-uniform lesion can be seen in Fig. 1c.

### Simulation of projection data

Phantoms of activity distribution and attenuation were generated using STIR software for tomographic imaging [19, 20] to represent imaging of $^{99{\mathrm{m}}}$Tc using parameters based on the collimator information of a SPECT system (Anyscan Trio, Mediso, Laborcutca 3. H-1037 Budapest Hungary) with LEHR collimators. STIR was used to forward project the generated phantom datasets described above, including the effect of attenuation and with PSF modelling. Attenuation was modelled in the simulation using the “full” model in STIR [21]. Sinograms were generated without scatter. In addition to noise-free data, noisy datasets were also generated by using the STIR utility to add Poisson noise. Total counts (i.e. the sum of all projections) in the simple phantom and bone scan datasets were approximately $5 \times 10^{6}$ to reflect counts, and therefore noise levels, seen in typical clinical SPECT bone scan imaging. Total counts for Tektrotyd imaging datasets were set to approximately $22 \times 10^{6}$, again reflecting typical clinical studies.

The components of the simple geometrical phantom (i.e. the background elliptical phantom, and a range of lesions of different positions and sizes) were forward projected separately and the resulting sinograms combined. Projecting as separate components allowed the sinograms of the lesion data to be scaled to achieve the different TBRs detailed in Table 1. Projections of the simple phantom were generated based on a circular orbit with a radius of rotation of 26 cm and 120 projections onto a matrix of 128✕ 128 isotropic pixels, 4.0 mm$^{2}$ in dimension.

The generated XCAT phantoms and separate lesions were forward projected and the resulting sinograms combined as above. Simulation of the forward projection was performed using an elliptical orbit (major axis radius = 250 mm, minor axis radius = 175 mm) with 120 projections onto a matrix of $128 \times 128$ isotropic pixels, 4.4 mm$^{2}$ in dimension.

### Perturbation

Point sources, consisting of activity in a single voxel, were generated and sinograms simulated using STIR in the same way as the phantoms described above. These sinograms were then scaled such that the intensity of the reconstructed point source was less than 10% of the intensity of the underlying voxel in the reconstructed image [14]. Reconstructions of the non-perturbed data were subtracted from reconstructions of the perturbed data to produce an image of the reconstructed PSF, indicating the situation-specific resolution. For the elliptical cylinder phantom, the perturbation points were positioned in two different positions in the FOV; the isocentre and 8 cm from the isocentre. In the anthropomorphic phantoms, the perturbation points were positioned as centrally as possible within the lesions.

### Reconstruction

Reconstructions with and without the added perturbation point source were performed in the same way. Reconstruction was performed in STIR, using OSEM with 10 subsets and 20 iterations (200 updates in total). The “simple” attenuation correction option in STIR was used in the reconstruction which is not as accurate as the “full” model [21] as used in sinogram generation, in an attempt to align with the real-life clinical situation where attenuation correction may be imperfect. Resolution Modelling was not applied in the reconstruction due to the disadvantages such as ringing artefacts and increased variance outlined above.

### PSF characterisation

2D Gaussians were fitted to the PSF in each orthogonal plane using Python’s curve fitting option in the optimisation package [22]. Fitting was performed based on a local region of the difference image. The offset of the Gaussian was fixed at zero above background. The theta parameter, representing rotation of the 2D Gaussian from the horizontal axis, was fixed based on the known position of the point within the image (i.e. was zero for points on axes). FWHM values for the radial, tangential and axial directions were calculated based on the average of values from two planes. Uncertainties were approximated based on estimated covariance of the optimised parameters given by the Python curve fitting function.

The FWHM values measured in different imaging situations were compared to examine dependence on parameters including reconstruction iteration (including whether PSF changes between sequential update images were smooth or unpredictable), lesion size and TBR, position in FOV, and surrounding activity distribution.

### Assessment of PSF accuracy

An assessment of the accuracy of the perturbation-estimated PSF, independent of any effects due to the PVC algorithm used, was required. This was done by comparing reconstructed noise-free images with GT images convolved with perturbation-estimated PSF. The Root-mean-square Error (RMSE) was calculated over a local volume around the lesion as follows:

1 $$\begin{aligned} {\text {RMSE}} = \sqrt{\frac{1}{N}\sum _{i=1}^{N}{(R_{i} - C_{i})}^2} \end{aligned}$$

where *N* is the number of voxels in the local volume, $R_{i}$ is the voxel value at position *i* in the reconstructed image, and $C_{i}$ is the voxel value at position *i* in an image of the GT convolved with the perturbation-estimated PSF. The RMSE was also calculated for reconstructed images compared with the GT convolved with a non-case-specific PSF. For this work, the non-specific PSF used was one measured at the centre of the FOV. This non-specific PSF was chosen since this may be the only position where the PSF is measured in practice.

### Partial volume correction with the single target correction method

**Fig. 2:**
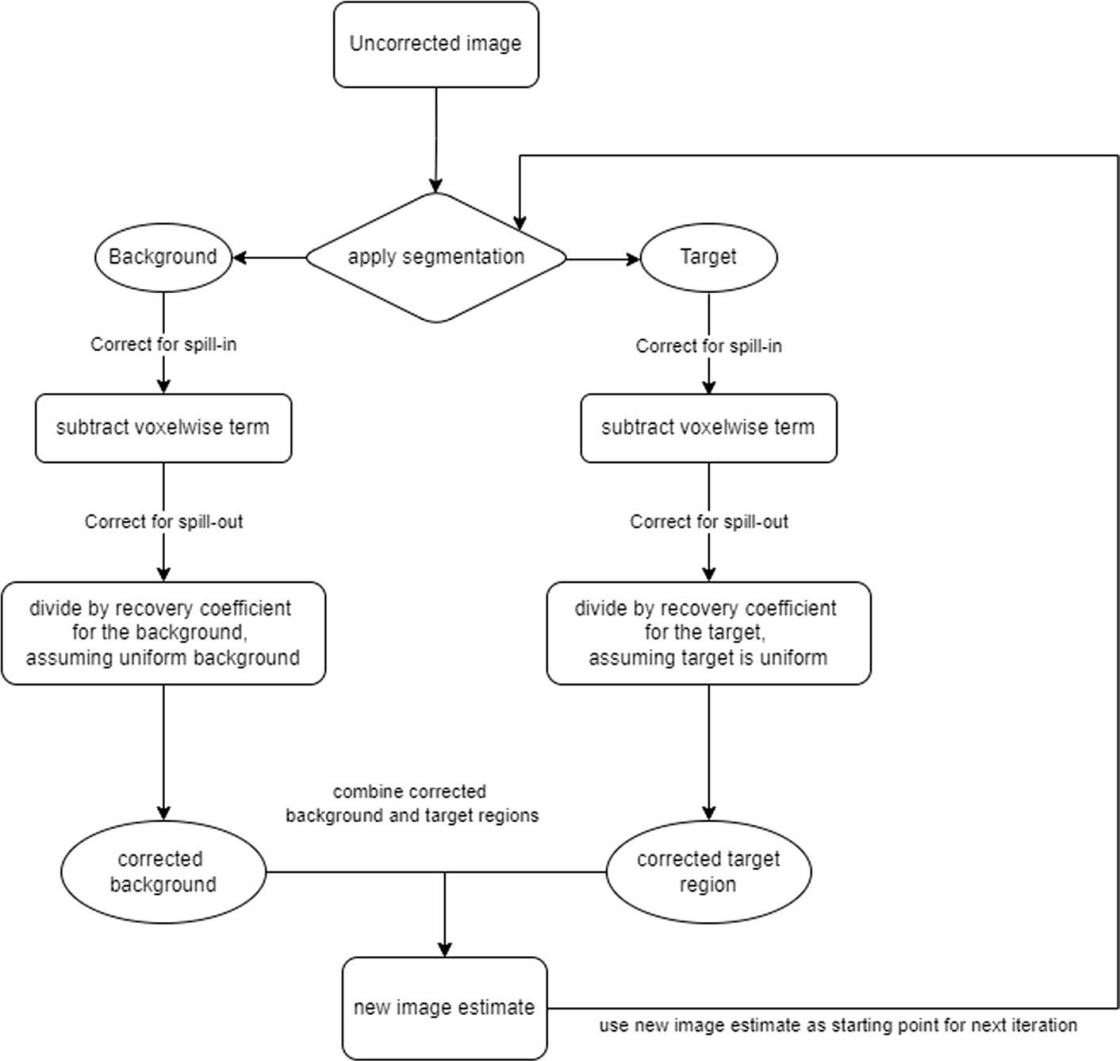
Flow chart demonstrating the steps involved in the iterative Single Target Correction (STC) partial volume correction method

PVC was applied to the reconstructed datasets using the Single Target Correction (STC) method, first proposed by Erlandsson and Hutton [23], also known as ‘Single Region Voxelwise’ correction. This method is well described by Sari et al. [24]. A short explanation will be given here and the algorithm is shown as a flow diagram in Fig. 2.

#### The single target correction method (STC)

Application of STC involves segmenting a single region of interest (aka the target), and corrections for spill-in and spill-out are applied to both the region and the surrounding background. Requirements for this method include knowledge of the PSF and segmentation of the volume of interest.

The STC algorithm is outlined in the following stages:

- Correcting the target for spill-in (subtracting a voxelwise term)
- Correcting the target for spill-out (by dividing by a recovery coefficient term for the target, and assuming that the target is uniform)
- Correcting the background for spill-in (subtracting a voxelwise term)
- Correcting the background for spill-out (dividing by a recovery coefficient term for the background, assuming uniform background)

These stages produce a new image estimate, which can be used as input to perform another iteration of the correction, i.e. repeating the above four steps.

One advantage of the STC method in the oncology setting is that segmentation is required only for a single region, as opposed to requiring segmentation of the entire image as per other segmentation-based PVC methods which have been published. In addition, the STC method returns voxelwise corrected images, as opposed to simply regional mean value data. Corrected images, e.g. with improved edge definition, may be useful in visual lesion detection, provided that segmentation is performed accurately.

In this work, STC was applied by segmenting the lesion based on the generated image. Ten STC iterations were used and a non-negativity constraint was imposed. The application of STC was tested in this work using a range of STC iterations for both the simple phantom and the XCAT phantom. A sufficient number of iterations in both cases was found to be 10, in agreement with previous work [24].

### Lesion quantification using regional mean values

Regional mean values (RMVs) of the lesion before and after PVC were assessed. PVC was performed using STC with the perturbation-estimated PSF. PVC was also performed using the non-specific PSF, as described above, in order to study the effect of PSF on quantification.

Application of PVC to XCAT data involved first resampling the emission image ($128 \times 128$ voxels) to match the Ground Truth image ($256 \times 256$ voxels), prior to PSF estimation, and before segmentation.

## Results

### Perturbation for PSF measurement

#### Variation with reconstruction update and position in FOV

**Fig. 3:**
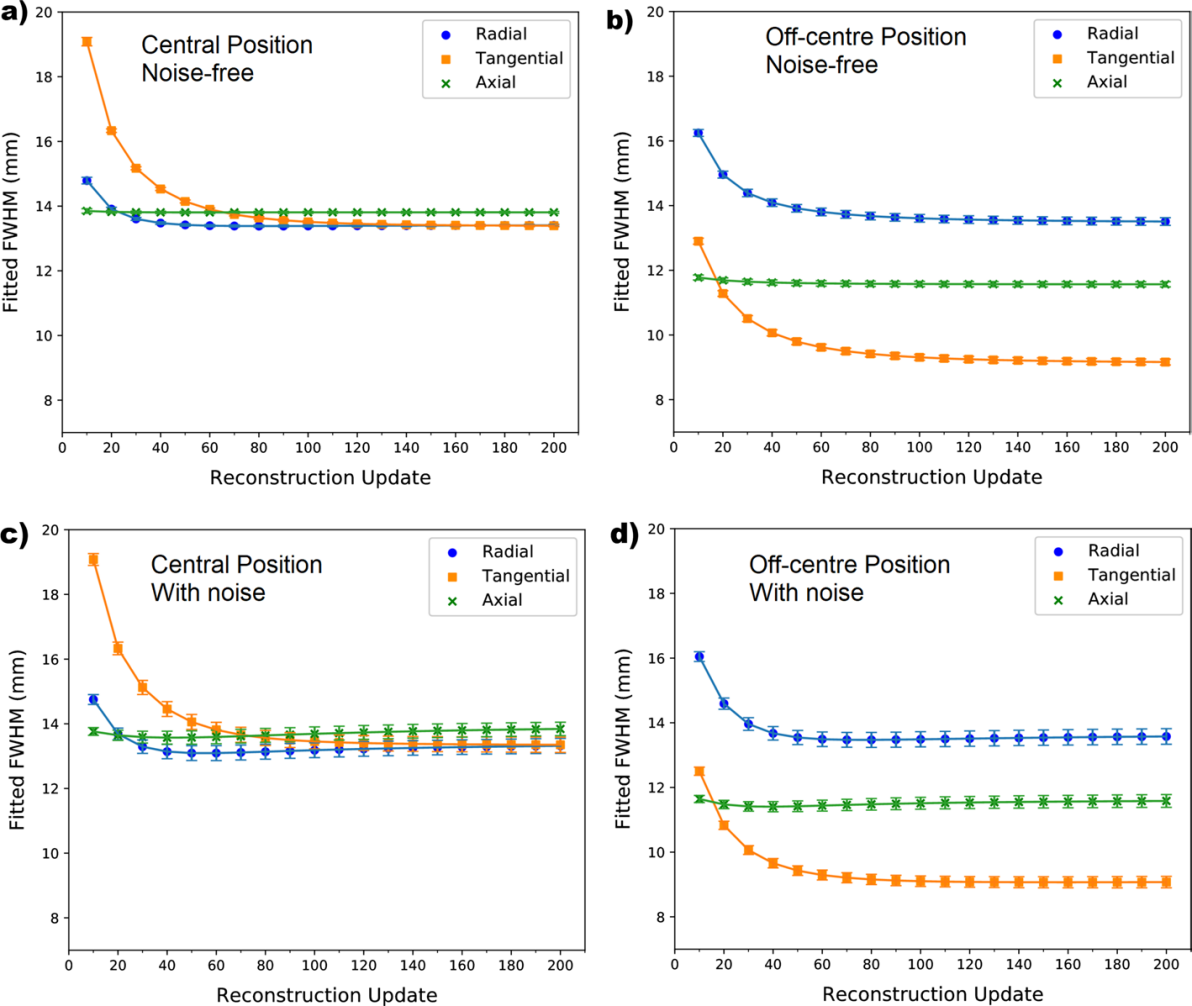
FWHM measurements in each direction vs reconstruction update number (10 updates = 1 full iteration) measured in **a** central lesion without noise, **b** off-centre lesion without noise, **c** central lesion with noise and **d** off-centre lesion with noise. The perturbation point was positioned in the centre of a 36 mm radius lesion and the lesion TBR was 10 in all cases shown. Error bars relate to the standard deviation in the fitted FWHM parameters but are too small to be seen clearly on noise-free data

As expected, FWHM measurements depended on the direction of measurement (radial, tangential and axial) and were spatially variant, i.e. depended on the location of measurement in the FOV. Figure 3 demonstrates FWHM measurements for noisy and noise-free data, for different reconstruction updates, measured in the simple geometrical phantom using a point source positioned centrally within a 36 mm radius lesion. The lesion was positioned either at the centre of the FOV (Fig. 3a, c) or 8 cm away from the isocentre, along the horizontal axis (Fig. 3b, d).

At 200 updates, the FWHM values in each direction depend on the position of measurement in the FOV. In addition, the PSF measured at the central point is roughly isotropic (the radial, tangential and axial FWHM values are within 3% of 13.4 mm in both noisy and noise-free data). The PSF at the off-centre position is anisotropic; the radial FWHM is $13.51 \pm 0.11$ mm, the axial is $11.57 \pm 0.08$ mm, and the tangential is $9.16 \pm 0.09$ mm for noise-free data. Measurements from noisy images of the off-centre point at 200 iterations are within 1 standard deviation (SD) of the noise-free FWHM measurements in each direction.

#### Variation with lesion contrast and size

**Fig. 4:**
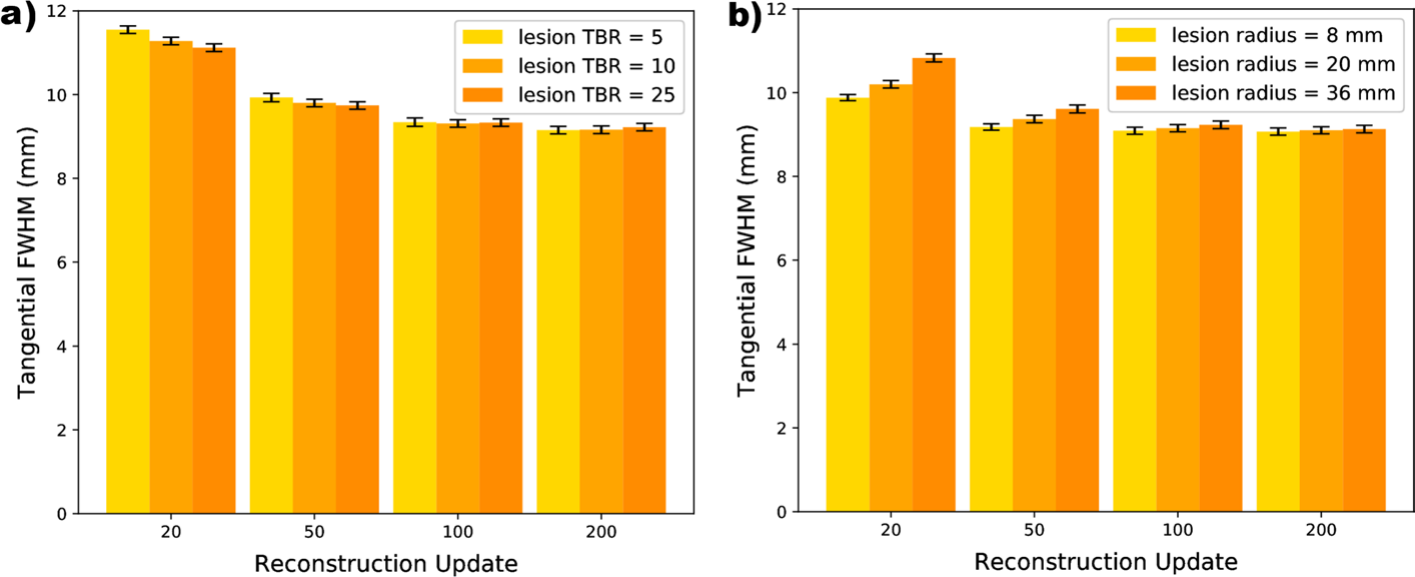
Graphs demonstrating the effect of lesion parameters of **a** lesion contrast and **b** lesion size on tangential FWHM measurements for selected reconstruction updates in noise-free images of the simple elliptical phantom. The point of measurement was in a lesion, 8 cm from the centre of the FOV

FWHM dependence was noted when measurements were made in lesions of different TBR, and different size. This is demonstrated for noise-free data in the simple elliptical phantom in Fig. 4, where Fig. 4a shows the effect of varying lesion contrast, and Fig. 4b shows the effect of varying lesion radius. However, this difference was observed only for images reconstructed at less than 100 OSEM updates. For images reconstructed with more updates, the FWHM measurements were virtually independent of lesion size and contrast (varied by less than 2.0% in noise-free data between all lesion sizes and contrasts at the same position for images reconstructed with 200 updates).

In noise-free data, for 20 updates, tangential FWHM measurement was found to vary from $11.12 \pm 0.09$ to $11.55 \pm 0.09$ mm depending on the lesion TBR (the difference from the TBR = 10 measurements is greater than 2 SDs for both of the other contrast settings measured). Beyond 100 updates the tangential FWHM measurement was 9.3 mm (within 1 SD) for all lesion contrasts, and for 200 updates the tangential FWHM was 9.2 mm (within 1 SD) for all lesion contrasts.

Similarly, noise-free data demonstrated increased variability in FWHM for different lesion sizes when measurements were made in images reconstructed with fewer updates. Measurements made in images reconstructed with 100 updates, or more, were all within 1 SD of each other.

#### Effect of point position within a lesion

The effect of point position within a lesion was studied in the simple cylindrical phantom. Reviewing the noise-free FWHM measurements demonstrated that the tangential FWHM measurements differed for different point positions relative to the lesion centre. Compared with the point at the centre, the point at the edge of the lesion had comparatively higher tangential FWHM values at early iterations (below about 100 updates), but by 200 updates, the values agreed well. However, for the point outside the lesion edge the tangential FWHM was still higher than the measurement made at the centre of the lesion for all image updates. Adding noise to these images resulted in increased uncertainty in the PSF fitting, resulting in larger errors.

#### Variation with surrounding activity distribution

FWHM measurements in lesions within the anthropomorphic datasets demonstrated that absolute FWHM values in each direction are all smaller than those measured at the central position in the simple phantom dataset.

This superior resolution can be explained by the closer proximity to the simulated detector face due to the lesion position being further from the centre of the FOV and due to the non-circular orbit. Adding Poisson noise had only a very small effect on the measured FWHM values.

**Fig. 5:**
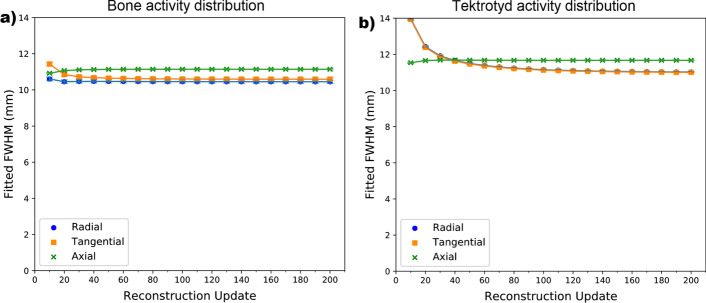
FWHM measurements in each direction vs reconstruction update number as measured in uniform lesions within anthropomorphic phantoms for noise-free data in an XCAT activity distribution simulating **a** a bone scan and **b** a Tektrotyd scan. The perturbation point was positioned in the centre of each spherical lesion. The bone scan lesion was positioned within the femoral head, adjacent to the bladder, and the Tektrotyd lesion was positioned within normal liver

A dependence on the number of reconstruction updates used in the image was observed, reflecting the finding in the simple dataset. However, one difference between datasets is that a relatively constant FWHM value is reached at a lower number of updates compared with what was observed for the simple phantom as shown in Fig. 5, compared with Fig. 3. Further investigation demonstrated that this difference was not due to the difference in orbit (circular vs non-circular), or lesion size, or lesion contrast.

A possible variation in activity distribution, commonly seen in clinical studies, which may vary between sequential scans, is the presence or lack of activity within the bladder. A simulation of the bone scan like activity distribution was performed with a bladder activity 15 times that of normal bone. Comparing these data to the zero activity bladder results shows only a very small difference (less than 1.7% between FWHM values) except for images reconstructed with fewer than 50 updates where up to 6.4% difference in FWHM value was measured.

#### Checking accuracy of perturbation-estimated PSF

The accuracy of the perturbation-estimated PSF data was assessed by evaluating the difference between reconstructed images and ground truth images convolved with a PSF. The difference was expressed as the RMSE.

**Fig. 6:**
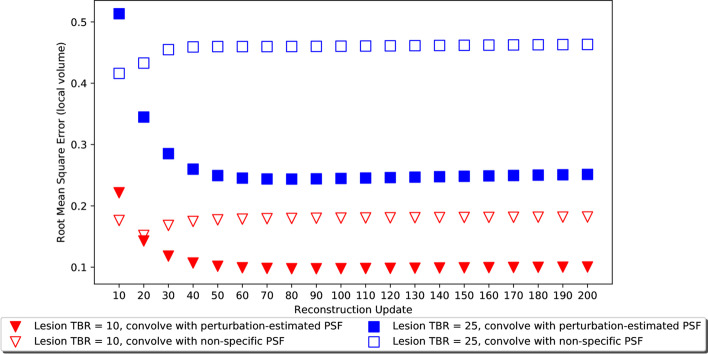
RMSE results for the 36 mm radius lesion, positioned 8 cm from the isocentre in the noise-free simple phantom. Results are shown for two different contrast settings, for perturbation-estimated PSF and a non-specific PSF

A consistently lower RMSE was demonstrated when convolution was performed using the perturbation-estimated PSF, compared with convolution using the non-specific PSF. This was the case for all images except those reconstructed with fewer than 20 updates. The RMSE assessment was performed for a range of lesion diameters and lesion contrasts. A subset of results is presented in Fig. 6. Higher contrasts and larger lesions resulted in higher absolute values of RMSE, as expected.

RMSE results for lesions within anthropomorphic datasets demonstrated a similar pattern; lower RMSE for convolution of the perturbation-estimated PSF for all but the lowest number of reconstruction updates.

### PVC results

#### Simple phantom

Applying PVC with the STC algorithm, using a perturbation-estimated PSF, resulted in more accurate lesion regional mean values than uncorrected data. This was the case for all reconstruction updates, for all lesion sizes, positions and lesion TBRs examined.

**Fig. 7:**
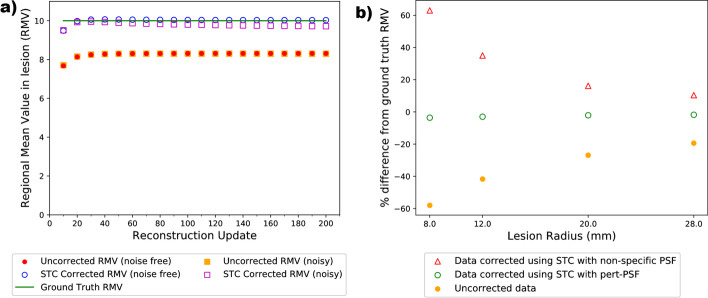
**a** Regional mean values (RMVs) measured for a lesion in the simple cylindrical phantom with 36 mm radius, TBR = 10, positioned centrally in FOV, for different image reconstruction update numbers. Note that the uncorrected noise-free and noisy datasets overlay each other. **b** Deviation in RMV from ground truth (in %) following STC with perturbation-estimated PSF or the non-specific PSF, for off-centre lesions and a range of lesion sizes. All lesions had TBR=10, and all images used 200 reconstruction updates

Figure 7a shows corrected and uncorrected regional mean values for a 36 mm radius lesion (TBR = 10) positioned at the centre of the FOV. Noisy and noise-free RMVs measured in uncorrected images were very similar, as expected. Uncorrected RMVs were an underestimate of the Ground Truth by between 17 and 23%. When PVC was applied to noise-free images, using a perturbation-estimated PSF, corrected RMVs are within 0.7% of ground truth in images reconstructed with more than 40 updates (within 0.4% for more than 100 updates). For noisy data, some reduction in accuracy is seen compared with noise-free data. However, corrected noisy RMVs are still within 2.8% of Ground Truth for images reconstructed with more than 20 updates.

Figure 7b demonstrates the deviation of the RMV from GT before and after applying PVC with STC for a range of lesion sizes within the simple phantom. The results are shown for noise-free images reconstructed with 200 updates, for off-centre lesions with TBR = 10 and a range of lesion sizes. Uncorrected data demonstrated underestimation of the RMV by between 19.4 and 58.0% compared with the ground truth. The largest deviation from ground truth was observed for the smallest lesion—as expected since smaller lesions are more affected by the PVE. Following the application of STC with the perturbation-estimated PSF, the corrected RMV agreed with the ground truth to within 3.6% for all lesion sizes. The residual post-correction bias was lower for larger lesions compared with the smallest lesions investigated. RMVs following the application of STC using a non-specific PSF were also calculated. The non-specific PSF used was wider than the perturbation-estimated PSF, and was therefore expected to over-correct the RMV. STC using this non-specific PSF overestimated the RMV compared with the ground truth by between 10.4 and 63.0%. The largest overestimation was observed for the smallest lesion.

**Fig. 8:**
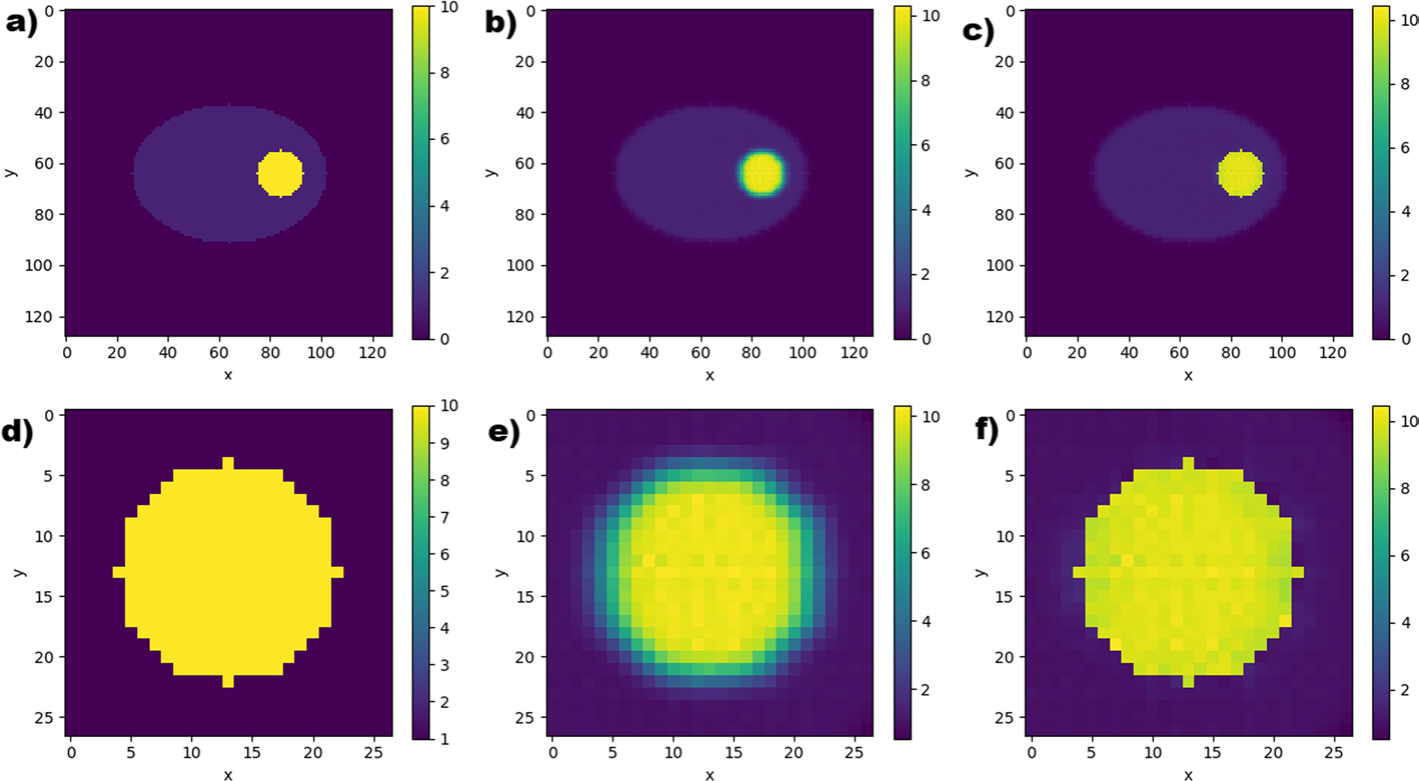
**a**–**c** from left to right; ground truth, uncorrected and corrected images, in the transaxial plane, through the centre of a 36 mm radius lesion within the simple phantom using noise-free data. **d**–**f** from left to right; local region around the lesion, zoomed in, on ground truth, uncorrected and corrected data

Example post-STC images are shown in Fig. 8c, f demonstrating that STC produces images with improved edge definition around the lesion of interest.

#### Anthropomorphic phantoms

**Fig. 9:**
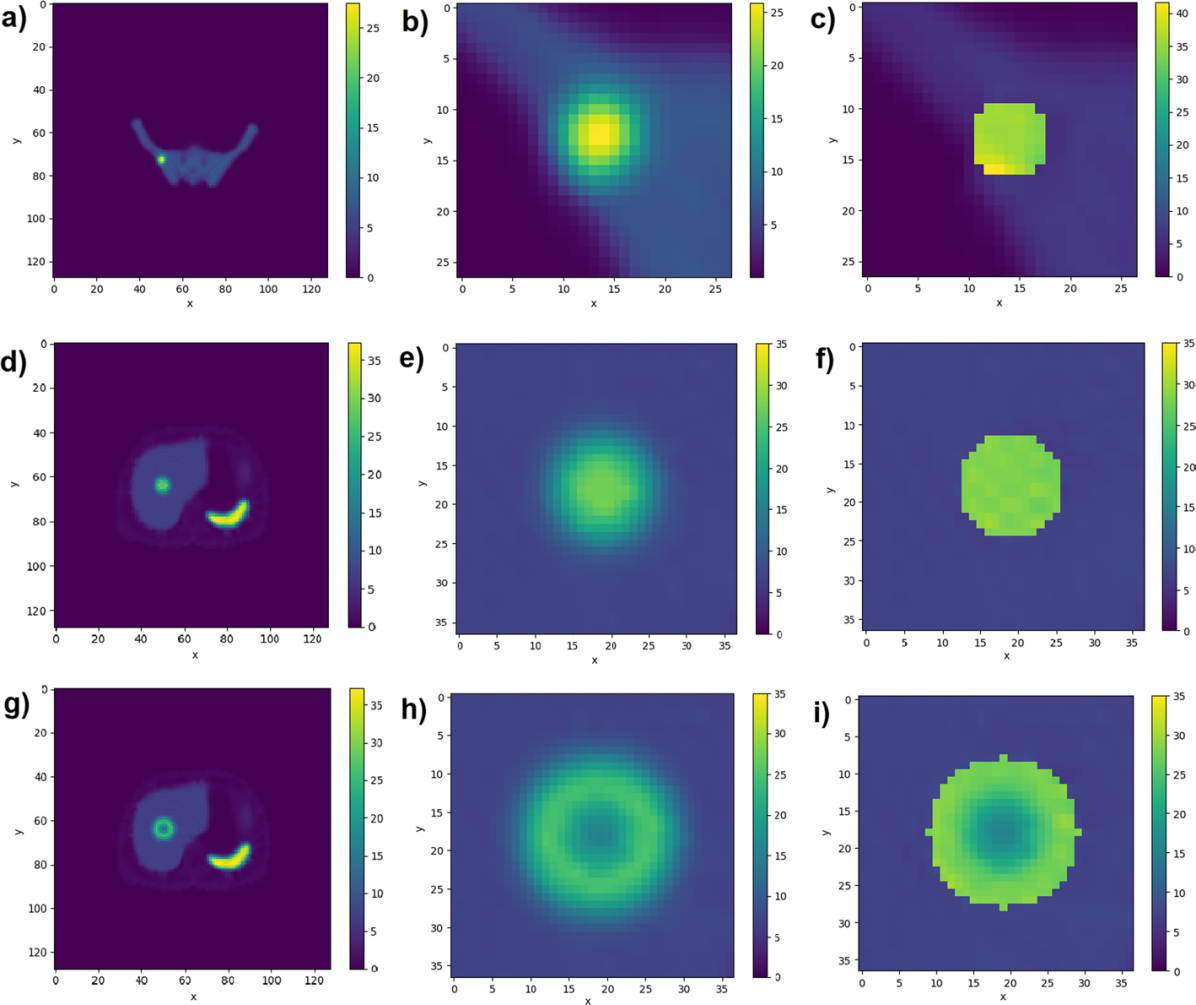
Noise-free images, in the transaxial plane, of the anthropomorphic phantom datasets. From left to right these show; uncorrected images reconstructed with 200 updates, the local region around the lesion in the uncorrected image, and post-PVC images after correction with 10 STC iterations. **a**–**c** The pelvis region of the bone scan phantom. **d**–**f** A uniform lesion in the liver of the Tektrotyd phantom. **g**–**i** The non-uniform lesion in the liver of the Tektrotyd phantom

Images of uncorrected and corrected transaxial slices through the centre of lesions in different positions within XCAT anthropomorphic phantoms are shown in Fig. 9. The top row shows the pelvis region of the bone scan phantom, the middle row shows a uniform lesion in the liver of the Tektrotyd phantom, and the bottom row shows the non-uniform lesion within the liver of the Tektrotyd phantom. As with the simple phantom, STC correction improves the edge definition, and also the quantitative accuracy.

For the bone phantom lesion shown in Fig. 9a–c, the RMV as measured from the uncorrected image underestimates the true value by between 47.7 and 50.6%, depending on the reconstruction update number. Applying PVC using the perturbation-estimated PSF and 10 STC updates resulted in corrected RMV within 1.6% of the ground truth for images reconstructed with more than 100 reconstruction updates. Applying PVC using a non-specific PSF resulted in an overestimation of the lesion RMV by up to 61.1%.

For the uniform Tektrotyd liver lesion shown in Fig. 9d–f, the RMV measured from the uncorrected image underestimates the true value by between 26.6 and 35.6%, depending on the reconstruction update number. For noisy data, the true value was underestimated by 26.8–36.5%. Applying PVC using the perturbation-estimated PSF and 10 STC iterations resulted in corrected RMV within 1.3% of the ground truth for images reconstructed with more than 100 reconstruction updates for both noisy and noise-free data. Another uniform lesion, in the same position but with a higher TBR, underestimated the true value by 29.7–36.8% for noise-free data and between 30.3 and 37.4% for noisy data. Corrected RMVs for both noisy and noise-free data were within 1.4% of the true value.

For the non-uniform Tektrotyd liver lesion shown in Fig. 9g–i, uncorrected RMVs were between 19.1 and 25.3% for noise-free data and between 20.3 and 26.1% for noisy data. Following PVC, the RMVs were within 1.9% for both noisy and noise-free data.

**Fig. 10:**
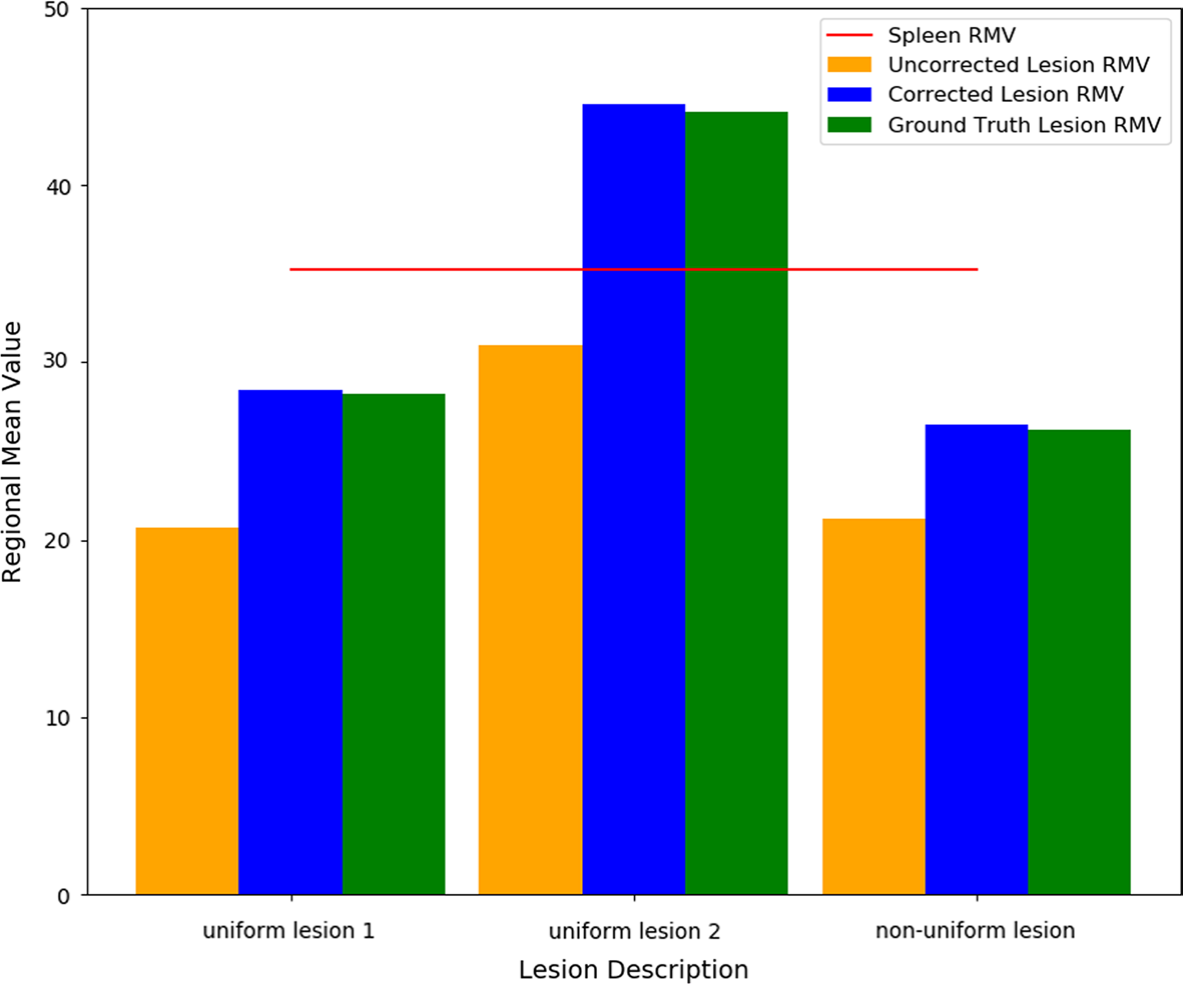
Regional mean values before and after STC correction, compared with Ground Truth, for noise-free data and 200 reconstruction updates, for the three different liver lesions investigated within the Tektrotyd XCAT dataset. The value for the spleen is also demarcated with a line demonstrating that the uncorrected RMV of uniform lesion 2 is lower than the spleen, but the ground truth and post-STC corrected RMV is actually greater than the spleen

Pre- and post-correction RMVs from the noise-free Tektrotyd datasets are shown in Fig. 10 for 200 reconstruction updates and 10 STC iterations demonstrating the improvement in RMV accuracy compared with ground truth for corrected data.

## Discussion

This work has shown the strong variation in PSF with location in FOV for SPECT imaging (Fig. 3). In addition, the assumption of an isotropic PSF is also inappropriate in most positions within the FOV. This indicates that accurate application of any PVC algorithm requiring information on the PSF requires a case-specific measurement.

One factor included in the ‘case-specific’ description is the number of updates used in the reconstruction. The PSF was found to depend on the reconstruction update number (Fig. 3). In each case investigated with the simple phantom, it takes around 100 reconstruction updates (10 OSEM iterations with 10 subsets) for the FWHM values in each direction to become roughly constant with reconstruction update number. Even at 200 updates, the shape of the plot suggests that the FWHM measurements could continue to change with reconstruction update number—in particular the tangential direction at the off-centre position.

Further support to a potential recommendation to ensure sufficient reconstruction updates are used for quantitative images is given in the results of the experiment comparing zero and non-zero activity within the bladder of the XCAT phantom. Here, a difference was seen when comparing otherwise identical images when images were reconstructed with fewer than 50 updates. For higher updates, the perturbation-estimated PSF was virtually independent of the presence of activity within the bladder.

The results presented here indicate that factors such as lesion contrast and intensity can also impact the PSF estimation if a low number of reconstruction updates is used (Fig. 4). The range of reconstruction updates this applies to include reconstruction settings typically used in clinical practice. For example, international guidance recommends 24–50 updates, and notes the need to balance noise and resolution [25]. Lesions may change in size and avidity throughout a course of treatment, and this can confound quantification since the extent of the PVE will vary due to these changes. Reconstructing with fewer than 100 OSEM updates may introduce variability in the PSF measurement which in turn may impact the accuracy of the applied PVC. However, further work would be required to assess the impact of varying PSF dimensions on lesion quantification.

Different FWHM variation behaviour was observed for the anthropomorphic phantoms when compared with the simple cylinder phantom. A possible explanation for this difference in behaviour is the difference in surrounding activity distribution. The lesions of interest within the anthropomorphic phantoms, in particular the XCAT bone phantom lesions, are surrounded by many more low activity voxels compared with the simple phantom lesions. An additional contribution may be the relative magnitudes of the FWHM values which are lower in the anthropomorphic phantom cases. This was explored further by simulating a lesion within a uniform ring, with the same external dimensions as the simple phantom, but with the inner volume set to zero voxel value. Simulation was performed using a circular orbit, as per the filled simple phantom. In this case, the lesion was positioned further from the centre of the FOV and the curve shapes were more similar to those observed in the XCAT data; reaching a relatively unchanging value at an earlier update number compared with the simple phantom. This supports the hypothesis that the difference in shape between the FWHM versus reconstruction update curves is due to the size of the PSF and/or the surrounding activity distribution.

Figure 3 shows that the standard deviation values on FWHM measurements are greater for earlier reconstruction updates. This suggests a higher level of uncertainty in the 2D Gaussian fits for images reconstructed with low numbers of iterations compared with more iterations. For increased confidence in the fit of the PSF, and to reduce the dependence of FWHM measurement on iteration number, this work suggests that at least 100 updates should be used for reconstruction.

Another relevant factor to consider, regarding typical clinical practice, is the matrix or voxel size used for acquisition and reconstruction. The normally accepted limit for sufficient sampling suggests that voxel dimensions should be less than 0.5 times the image resolution [26]. In the cases studied in this work, to reflect typical clinical practice, 4 mm or 4.4 mm voxels were used. Referring to Fig. 3, we observe FWHM values approaching approximately 8 mm. These measurements were based on a position 8 cm from the centre of the FOV, however for objects positioned closer to the collimator face, the resolution is expected to be superior and therefore voxel sizes may exceed the suggested limit.

Changing acquisition and reconstruction parameters will affect image appearance—possibly adversely. To decouple the problem of producing visually good images, for clinical reporting, and quantitatively accurate datasets there is an argument for reconstructing multiple different image sets. For example, reconstructing one set of images for visual interpretation and another with smaller voxels, reconstructed using more iterations which could be used for quantification. However, we must note that an adverse effect of increasing the number of OSEM iterations is an increase in image noise. The optimal reconstruction settings would need to balance the bias and variance for quantification.

Another factor relevant to the reconstruction, as mentioned in the introduction, is the use of Resolution Modelling. The data presented in this work have all been reconstructed without the use of RM. Had RM been used in the reconstruction, we may have expected to see some reduction in the underestimation of regional mean value measurements for some non-PVC data. However, this reduction in bias would depend on the size and intensity of the lesion and on other reconstruction parameters. Due to Gibbs artefacts, for some smaller lesions, data reconstructed with RM may demonstrate a maximum voxel value of greater than 100% of the ground truth [27]. The application of PVC using perturbation to data reconstructed using RM is not guaranteed to be reliably accurate since Gibbs artefacts would also make the assumption of a Gaussian PSF inappropriate. In addition, the assumption of constant PSF across the width of a lesion may no longer be appropriate due to increased enhancement (i.e. improved resolution) at the edges of an object compared with the centre.

The accuracy of corrected images depends on both the accuracy of the PVC method and the accuracy of the resolution measurement. Therefore, residual bias in regional mean values may not be solely due to limitations of the perturbation method—they may be due to limitations of the STC method. However, the RMSE analysis did demonstrate lower error when the image was convolved with a perturbation-estimated PSF versus a non-specific PSF, acting as a check of the suitability of the perturbation method independent of the PVC method.

One advantage of the STC method for partial volume correction is the production of a corrected image, rather than assuming uniformity across the lesion and assigning a single value across the region. Since some stages of the STC correction include voxelwise correction, the heterogenity of the lesion could be retained. This is demonstrated in Fig. 9, in particular the bottom row which shows the STC method applied to a non-uniform lesion with uptake pattern typical of a lesion with a necrotic core.

An activity distribution based on Tektrotyd studies was investigated as this may be an area of clinical practice where the accurate application of PVC could be important. The semi-quantitative Krenning score which assesses the uptake in lesions relative to the uptake in the liver and in the spleen [28] has been shown to correlate with outcome [29]. In general, lesions are assessed visually, however there is interest in reviewing these studies with quantitative SPECT [18]. To illustrate the impact of the PVE, and the importance of accurate application of PVC, we included a lesion in the Tektrotyd dataset with Ground Truth uptake higher than the spleen (corresponding to a Krenning score of 4). However due to the PVE, as show in Fig. 10, the uncorrected reconstructed image demonstrates a regional mean value higher than the liver but lower than the spleen (corresponding to a Krenning score of 3). The extent of the PVE depends on multiple factors including the size, contrast and position within the FOV. Accurate application of PVC would improve lesion and position independence when quantifying uptake. This is especially important as Tektrotyd studies can be used to monitor response to treatment where size and avidity of the same lesion is likely to change which, as noted above, can confound quantification. Accurate application of PVC could reduce the impact of this effect.

### Limitations

One limitation of this work is the fact that system models and software used for generating sinograms and for reconstructing were identical. This does not replicate the “real life” situation accurately. However, we performed some simulations with non-matched system models. From these simulations, the estimated PSF was found to depend on the system model used to simulate imaging of the point source (i.e. used to generate the sinogram of the perturbation source). A possible option to pursue in clinical practice may be to use a measured point source—ensuring that the geometry and conditions for generating the sinogram of the perturbation source exactly matched that of the acquisition of patient data. However, a significant disadvantage of this is the requirement to make a measurement at every possible position in the transaxial plane. In addition, the measurement would not match the patient-specific attenuation and scatter situation. There would be experimental errors also in the production of a point source. Therefore, work to assess the required accuracy of the system model would be beneficial to guide the assessment of the suitability of using a simulated perturbation source.

This digital phantom simulation study did not investigate other factors which may limit accuracy in the practical application of SPECT quantification. For example, in this work segmentation was performed directly from the generated image and was therefore accurate. While noisy datasets were produced, scatter was not modelled and so results here assume perfect scatter correction. Scatter correction methods are commonly accepted to be reasonably accurate for $^{99{\mathrm{m}}}$Tc SPECT, however may not be perfect as assumed in the current simulation. While an imperfect attenuation correction was applied in the reconstruction, we acknowledge that other factors which limit the accuracy of attenuation correction in real clinical data are not replicated here. In reality, the accuracy of attenuation correction based on CT images may be limited by issues related to scaling Hounsfield Unit values to the energy of the SPECT photons, mis-registration and image noise. Real-life scatter and attenuation conditions may result in a reduction in the accuracy of the perturbation measurement and PVC applied to clinical data. Further work should involve testing the perturbation method and PVC algorithm on clinical datasets—potentially utilising Monte Carlo simulation with synthetically added lesions to provide a ground truth for the lesion. For translation into clinical practice, in addition to testing the suitability of a simulated perturbation source as mentioned above, it would be important to include the effect of segmentation, registration, scatter and attenuation correction into the overall assessment of quantitative accuracy. Again, further work on error propagation within SPECT quantification, and estimation of the resulting uncertainty, would be important for the evaluation of an optimal PVC method. For clinical data, uncertainty analysis could be performed by following the EANM guidelines [30].

Note that some error may be introduced due to variation in the PSF across the distance of the lesion. This work assumes that, while the PSF may change significantly across the FOV, it does not change significantly across the local region around the lesion or object of interest. “Significantly” in this case refers to variations in the PSF that would impact the accurate application of PVC such that the result of quantification would be different. This is likely to be a reasonable assumption for small lesions, but may not be for larger objects, e.g. organs at risk. However, since larger objects will be less affected by the PVE in the first instance, there may be a trade-off in accuracy. Further assessment of this would be useful.

Referring to Fig. 9c, while the quantitative accuracy of the STC correction was good, and the corrected image had improved edge definition, the uniformity of the ground truth lesion was not reproduced in the corrected image. This merits further exploration.

Areas of further investigation around the subject of this work include, but are not limited to, an assessment of precision (i.e. evaluating uncertainties), and error propagation—including an assessment of the required accuracy of the system model used for sinogram generation/reconstruction. Data presented here were limited to the relatively simple imaging radionuclide, $^{99{\mathrm{m}}}$Tc. Further investigation would be required to test whether the application of the perturbation method can be generalised to other radionuclides such as those used for imaging in the context of theranostics (for example $^{123}$I or $^{111}$In), or those used with therapeutic intent (for example $^{90}$Y, $^{131}$I or $^{177}$Lu). The PSF of these radionuclides is likely to be wider and may include star artefacts due to high energy emissions and septal penetration. The perturbation method could potentially account for this, provided that the implementation does not make the assumption of a Gaussian PSF. Further investigation towards implementing perturbation on radionuclides other than $^{99{\mathrm{m}}}$Tc would be informative, perhaps utilising Monte-Carlo modelling to explore shapes and position-dependence of the PSF.

## Conclusion

Perturbation, using a single voxel point source, was applied to simulated phantoms replicating situations that may be encountered in clinical SPECT oncology imaging. Perturbation reliably estimated PSF values, appropriate for the specific situation, provided that a sufficient number of reconstruction iterations were used.

When PVC was applied using a PSF as estimated by perturbation, quantification of regional mean values was more accurate than non-corrected data, and was also more accurate compared with PVC applied using a non-specific PSF.

While further study is required in the field of PVC in oncology SPECT, this work demonstrates that using the perturbation technique to estimate the PSF and using this information in the application of PVC can improve quantitative accuracy. Improved quantitative accuracy in oncology SPECT would have benefits in multiple applications including diagnosis, monitoring disease progression, monitoring response to treatment, and radionuclide therapy dosimetry.

## Data Availability

The datasets generated and analysed during the current study are available from the corresponding author on reasonable request.

## Abbreviations

$$^{99{\mathrm{m}}}$$Tc: Technetium-99m
FOV: Field of view
FWHM: Full width at half maximum
GT: Ground truth
OSEM: Ordered subset expectation maximisation
PET: Positron emission tomography
PSF: Point spread function
PVC: Partial volume correction
PVE: Partial volume effect
RM: Resolution modelling
RMSE: Root-mean-square error
RMV: Regional mean value
SD: Standard deviation
SPECT: Single photon emission computed tomography
STC: Single target correction
STIR: Software for tomographic image reconstruction
TBR: Target to background ratio
XCAT: Extended cardiac-torso phantom
